# Orthorexia Symptoms in Adolescents: Associations with Sociocultural Attitudes Toward Appearance and Body Composition

**DOI:** 10.3390/children13101355

**Published:** 2026-10-05

**Authors:** Agata Kiciak, Wiktoria Staśkiewicz, Natalia Kuczka-Marek, Agnieszka Białek-Dratwa, Anna-Maria Sapała, Sylwia Jaruga-Sękowska, Marek Kardas, Renata Barczyńska

**Affiliations:** 1 Department of Food Technology and Quality Assessment, Department of Dietetics, Faculty of Public Health in Bytom, Medical University of Silesia in Katowice, ul. Jordana 19, 41-808 Zabrze, Poland; wstaskiewicz@sum.edu.pl (W.S.); mkardas@sum.edu.pl (M.K.); 2 Doctoral School, Medical University of Silesia in Katowice, 40-055 Katowice, Poland; d201362@365.sum.edu.pl; 3 Department of Human Nutrition, Department of Dietetics, Faculty of Public Health in Bytom, Medical University of Silesia in Katowice, ul. Jordana 19, 41-808 Zabrze, Poland; abialek@sum.edu.pl; 4 Department of Dietetics and Food Science, Faculty of Science, Natural and Technical Sciences, Jan Długosz University in Częstochowa, Aleja Armii Krajowej 13/15, 42-200 Czestochowa, Poland; am.stelmach@ujd.edu.pl (A.-M.S.); r.barczynska-felusiak@ujd.edu.pl (R.B.); 5 Department of Health Promotion, Faculty of Public Health in Bytom, Medical University of Silesia in Katowice, ul. Piekarska 18, 41-902 Bytom, Poland; sjaruga@sum.edu.pl; 6 Food Industry School, Franciszkańska 13, 41-819 Zabrze, Poland

**Keywords:** orthorexia nervosa, adolescent mental health, sociocultural pressure, body image

## Abstract

**Highlights:**

**What are the main findings?**

- Orthorexic symptom severity in adolescents was positively associated with appearance-related sociocultural pressure, athletic internalization, and appearance-related information.
- After adjustment for sociocultural factors, percentage body fat, sex, and age, no individual predictor showed an independent association with orthorexic symptom severity at the conventional significance threshold.

**What are the implications of the main findings?**

- The findings highlight the relevance of appearance-related sociocultural influences when investigating orthorexic symptoms in adolescents, while indicating that these relationships are complex and should not be interpreted as causal.
- Longitudinal studies using adolescent-validated orthorexia measures and direct assessments of nutrition- and appearance-related digital exposure are needed to clarify potential pathways and inform future prevention strategies.

**Abstract:**

Background/Objectives: Orthorexic symptoms in adolescence may be associated with sociocultural appearance influences, but their relationship with objective body composition remains unclear. This study examined associations between orthorexic symptoms, sociocultural attitudes toward appearance, and body composition in adolescents. Methods: This cross-sectional study included 287 Polish secondary-school students aged 14–18 years. Orthorexic symptoms were assessed using the Düsseldorf Orthorexia Scale (DOS), sociocultural attitudes using the Sociocultural Attitudes Towards Appearance Questionnaire-3 (SATAQ-3), and body composition by bioelectrical impedance analysis. Correlation and multiple linear regression analyses were performed. Results: DOS scores correlated positively with SATAQ-3 pressure/internalization (r = 0.407, *p* < 0.001), athletic internalization (r = 0.330, *p* < 0.001), and Information (r = 0.385, *p* < 0.001). These associations remained robust in Spearman sensitivity analyses. The regression model including four SATAQ-3 subscales, percentage body fat (PBF), sex, and age explained 19.2% of DOS-score variance (adjusted R^2^ = 0.172; F(7.279) = 9.49, *p* < 0.001). Pressure/internalization (β = 0.190, *p* = 0.053) and Information (β = 0.162, *p* = 0.060) did not reach conventional statistical significance. The remaining predictors were not independently associated with DOS scores. Conclusions: Orthorexic symptom severity was associated with appearance-related sociocultural variables at the bivariate level, but individual predictors were not independently significant in the adjusted model. These findings identify sociocultural factors as relevant correlates without establishing causality or excluding a role for body composition. Longitudinal studies using adolescent-validated measures and direct assessments of digital exposure are needed.

## 1. Introduction

Orthorexic symptoms refer to a rigid preoccupation with eating perceived as healthy, pure, or high quality and to dietary rules that may impair daily functioning [1,2,3]. Although orthorexia nervosa is discussed as a possible eating-related disorder, its diagnostic status remains unsettled. Orthorexic concerns may overlap with other forms of eating pathology, but motives centered on healthfulness, food purity, quality, and control are not reducible to weight or appearance dissatisfaction.

During adolescence, body image and eating behavior develop within peer, family, and media environments. Repeated exposure to idealized thin or muscular bodies, appearance comparison, and perceived pressure may encourage internalization of appearance standards and restrictive behaviors [4,5,6,7,8,9,10,11]. Studies in adolescents have also examined associations of social media comparison and appearance-ideal internalization with body dissatisfaction and well-being [12,13,14]. These processes are relevant to disordered eating generally, but in orthorexia they should be considered one possible pathway among psychological traits, health beliefs, family influences, and broader social contexts.

The SATAQ-3 assesses perceived appearance pressure, internalization of appearance ideals, and the use of media as a source of appearance-related information [15,16]. It does not measure exposure to healthy-eating or nutrition content specifically. Accordingly, an association between SATAQ-3 and DOS scores would indicate an appearance-related sociocultural association, not proof that health-oriented digital content causes orthorexic symptoms. A recent systematic review nevertheless suggests that idealized body images and fitness lifestyles on social media may intersect with symptoms of orthorexia nervosa and muscle dysmorphia, supporting careful study of this broader context [17].

Objective body composition provides information that is distinct from perceived body image. Adolescents with similar BMI, body-fat percentage, or muscle mass may evaluate their bodies differently, and perceived discrepancies from appearance ideals may be more closely related to eating cognitions than the measured physical characteristics themselves [18,19,20]. Simultaneous assessment of sociocultural variables and body composition can therefore clarify whether each shows an independent association with orthorexic symptom severity.

Sex differences also require cautious interpretation. Girls and boys may encounter different appearance ideals, while the SATAQ-3 was developed primarily around thin-ideal internalization and may not represent muscularity-oriented pressures equivalently [15,16,21]. Without formal measurement-invariance testing, observed score differences cannot be assumed to reflect greater susceptibility in either sex.

Digital environments involve more than passive exposure to idealized images. Appearance-related social media consciousness describes ongoing awareness of how one may appear to an online audience [22], while the identity-bubble framework emphasizes social identification, homophily, and information filtering in adolescent online networks [23]. Neither construct was measured in the present study; they are introduced only as directions for future research rather than explanations of the current findings. Reviews have also examined social media and food advertising in relation to adolescents’ food choices [24,25].

The aim of this study was to examine associations between orthorexic symptom severity, appearance-related sociocultural attitudes, and objectively measured body composition in adolescents. We also examined descriptive sex differences and whether SATAQ-3 subscales and a selected body-composition indicator were independently associated with continuous DOS scores after adjustment for sex and age.

## 2. Materials and Methods

### 2.1. Participants and Procedure

The study was conducted between January and February 2025 using questionnaires and body-composition assessment by bioelectrical impedance analysis (BIA). Participants were secondary-school students aged 14–18 years. Of 434 students invited, 140 declined participation. Of the 294 students who entered the study, 7 were excluded because of incomplete questionnaire or measurement data. The final analytical sample comprised 287 adolescents (66.1% of those invited), including 182 females (63.4%) and 105 males (36.6%). All participants were recruited from secondary schools in the Silesian region of Poland, representing a relatively homogeneous Central European population. The final dataset contained complete data for all variables used in Table 1, Table 2, Table 3, Table 4 and Table 5 and in the regression analysis.

The sampling procedure was purposive and non-random (convenience sampling). Students present on the day of data collection were invited to participate and provided informed consent (for minors, parental or legal guardian consent was obtained). Participants received instructions on how to complete the questionnaires and accessed the survey electronically via a QR code. Questionnaire data were collected using the Google Forms platform. Inclusion criteria were age 14–18 years, enrollment in secondary school, and no contraindications to body composition assessment using BIA. The exclusion criterion was incomplete questionnaire or measurement data.

Two validated psychometric instruments were employed: the Sociocultural Attitudes Toward Appearance Questionnaire-3 (SATAQ-3) and the Düsseldorf Orthorexia Scale (DOS).

#### 2.1.1. Body Composition Analysis

Body height was measured to the nearest 0.1 cm using a portable stadiometer. Measurements were performed with participants standing barefoot, with the heels, buttocks, and upper back in contact with the vertical surface of the stadiometer and the head positioned in the Frankfurt plane. Participants stood upright with arms hanging freely alongside the body, and the measurement was recorded at full extension during a gentle inhalation.

Body mass and body composition were assessed by bioelectrical impedance analysis (BIA) using an InBody 270 analyzer (InBody Co., Ltd., Seoul, Republic of Korea). Measurements were performed according to the manufacturer’s protocol under standardized conditions: participants were barefoot, wearing light clothing, without metal objects, after voiding, and refrained from food intake and vigorous physical activity for at least 2–3 h prior to assessment. During the measurement, participants stood still on the device platform with their feet placed on the designated electrodes and held the hand electrodes with the upper limbs slightly abducted, following the device instructions.

The following parameters were obtained: body mass (kg), body mass index (BMI, kg/m^2^), skeletal muscle mass (kg), and percentage body fat (%). BMI was calculated as body mass (kg) divided by height squared (m^2^).

BMI values were interpreted according to the World Health Organization (WHO) classification; BMI was also assessed using age- and sex-specific percentile charts designed to evaluate the height and nutritional status of children and adolescents [26,27].

#### 2.1.2. SATAQ-3

Sociocultural attitudes toward appearance and the degree of internalization of appearance ideals were assessed using the Sociocultural Attitudes Toward Appearance Questionnaire-3 (SATAQ-3) developed by Heinberg and Thompson [15]. This widely used instrument evaluates the influence of sociocultural norms, particularly media messages, on attitudes and behaviors related to body image and physical appearance. The scale measures the extent to which individuals experience appearance-related social pressure and internalize culturally promoted standards of attractiveness.

The SATAQ-3 consists of 30 items rated on a 5-point Likert scale (1—“strongly disagree” to 5—“strongly agree”) and comprises four subscales: internalization-general, internalization-athlete, pressures, and information.

In the present study, the Polish adaptation of the SATAQ-3 developed by Izydorczyk and Lizińczyk was used, demonstrating good psychometric properties in adolescent and young adult populations [16]. In the present sample, the internal consistency of the SATAQ-3 subscales was satisfactory. Cronbach’s alpha coefficients were: internalization–general α = 0.87, internalization–athlete α = 0.78, pressures α = 0.84, and information α = 0.77.

Higher scores on the SATAQ-3 indicate a greater degree of internalization of sociocultural appearance ideals and perceived pressure related to appearance. The internalization–general subscale reflects the extent to which individuals adopt widely promoted appearance standards (e.g., thinness), whereas the internalization–athlete subscale captures the internalization of athletic and muscular ideals. The pressure subscale assesses perceived external pressure from media and social environment to conform to appearance norms. The information subscale reflects the extent to which individuals actively seek and use media sources as guidance for appearance-related standards. The information seeking subscale refers to the tendency to engage with and attend to appearance-related content in media.

Each subscale is interpreted independently, with higher scores indicating stronger endorsement of the respective sociocultural influence. The total score represents the overall level of sociocultural influence on body image.

#### 2.1.3. DOS

The severity of orthorexia symptoms was assessed using the Düsseldorf Orthorexia Scale (DOS) developed by Chard et al. [1]. The DOS is a screening instrument designed to evaluate eating behaviors characteristic of orthorexia nervosa (ON), defined as a pathological preoccupation with healthy eating and rigid adherence to dietary rules motivated by beliefs about the healthfulness of food. The scale suggests high internal consistency, good construct validity, and satisfactory test–retest reliability.

In population-based research, the 10-item version of the DOS is commonly applied as a unidimensional measure of orthorexic tendencies and for screening purposes. Items are rated on a 4-point Likert scale (1—“does not apply to me at all” to 4—“applies to me very much”), with no reverse-scored items. The total score is calculated as the sum of item responses (range: 10–40), with higher scores indicating greater severity of orthorexic symptoms.

For descriptive screening analyses, the proposed DOS thresholds were used: scores below 25, scores of 25–29, and scores of 30 or higher. These categories were treated as screening-based classifications of symptom severity, not diagnostic groups, because the proposed cutoffs were developed in adults and have not been validated specifically in Polish adolescents. The continuous DOS score was used in correlation and regression analyses.

In the present study, the Polish language version of the DOS was used, which has demonstrated good psychometric properties [2]. In the present study, the DOS demonstrated good internal consistency (Cronbach’s α = 0.82).

### 2.2. Ethical Aspects

All participants and their legal guardians were fully informed about the purpose of the study and assured of anonymity, and consent for data use was obtained prior to the commencement of data collection.

The study was conducted in accordance with the Declaration of Helsinki and was approved by the Bioethics Committee of the Medical University of Silesia in Katowice (protocol code: BNW/NWN/0052/KB1/20/23; approval date: 28 March 2023). Written informed consent was obtained from all participants and their legal guardians.

### 2.3. Statistical Analysis

Statistical analyses were performed using Statistica version 14.2 (TIBCO Software Inc., Palo Alto, CA, USA) and IBM SPSS Statistics version 30.0.0 (IBM Corp., Armonk, NY, USA). Continuous variables are presented as means and standard deviations (SD), and categorical variables as frequencies and percentages. Normality of distribution was assessed using the Shapiro–Wilk test.

Between-sex differences in continuous variables were analyzed using an independent-samples *t*-test or Mann-Whitney U test, as appropriate. Differences in categorical variables were evaluated with the chi-squared test. Comparisons across DOS screening categories were performed using the Kruskal-Wallis test. When the omnibus test was significant, pairwise comparisons were conducted using Dunn’s test with Holm adjustment.

Pearson correlation coefficients were used as the primary descriptive measures of linear association in the full sample (*N* = 287). Because several variables were non-normally distributed and observations at distributional extremes were retained, Spearman rank correlations were calculated as a sensitivity analysis for the associations between DOS scores and the four SATAQ-3 subscales. Multiple linear regression was conducted with continuous DOS score as the dependent variable. The initially planned eight-predictor specification included the four SATAQ-3 subscales, BMI, percentage body fat (PBF), skeletal muscle mass (SMM), and sex. Collinearity diagnostics showed substantial redundancy among the body-composition variables (VIF: BMI = 10.70, PBF = 9.96, SMM = 6.94), so this specification was not retained for interpretation of individual coefficients. The primary model included the four SATAQ-3 subscales, PBF as the single adiposity indicator, sex (female = 1, male = 0), and age as a developmental covariate. PBF was selected to represent adiposity while avoiding simultaneous inclusion of strongly overlapping BIA-derived measures. Collinearity was assessed using variance inflation factors (VIFs), tolerance values, and condition indices. Unstandardized B, standard error, standardized beta, 95% confidence intervals, *p* values, VIFs, and tolerances are reported. All 287 participants had complete data for the variables in the primary model; no missing-data procedure was required.

Two-sided statistical significance was set at *p* < 0.05. The full correlation matrix was treated as exploratory. As a multiplicity sensitivity analysis, the ten correlations between DOS and the other variables in Table 4 were also evaluated against a Bonferroni threshold of *p* < 0.005.

No prospective a priori power analysis was conducted before data collection. A retrospective sample-size calculation was therefore performed in G*Power version 3.1.9.7 for linear multiple regression (fixed model, R-squared deviation from zero). With alpha = 0.05, power = 0.80, a medium effect size (f-squared = 0.15), and eight planned predictors, the conventional minimum sample size was 109. The final sample of 287 exceeded this planning threshold; this later calculation is not presented as an a priori analysis.

Although the SATAQ-3 is widely used in adolescent samples, it was developed primarily around thin-ideal internalization and may not fully capture male-specific appearance ideals such as muscularity. Measurement invariance across sexes was not tested; therefore, between-sex comparisons were interpreted descriptively and with caution.

Observations at distributional extremes, including high BMI values, were checked and retained because they represented plausible biological variability rather than data-entry or measurement errors. Robustness to these observations and non-normality was evaluated through non-parametric group tests and the Spearman correlation sensitivity analysis described above.

## 3. Results

### 3.1. Participant Characteristics, Body Composition, and Psychosocial Measures

The general characteristics and body composition of the study population are presented in Table 1. The sample included 287 adolescents aged 14–18 years. No significant sex difference was observed for age. Males exhibited significantly higher total body water, fat-free mass, and skeletal muscle mass, whereas females had significantly higher body fat mass and percentage body fat. No significant sex differences were observed for WHR.

**Table 1:** Characteristics and body composition of the study participants (*n* = 287).

Variable	Total (*n* = 287)	Females (*n* = 182)	Males (*n* = 105)	*p*-Value
	X ± SD	X ± SD	X ± SD	
Age (years)	16.94 ± 1.29	17.06 ± 1.34	16.74 ± 1.17	0.052
Height (cm)	169.32 ± 8.87	164.88 ± 6.02	177.01 ± 7.70	<0.001 *
Weight (kg)	64.22 ± 15.12	59.43 ± 11.51	72.52 ± 16.98	<0.001 *
BMI (kg/m^2^)	22.27 ± 4.27	21.81 ± 3.78	23.08 ± 4.92	0.015 *
WHR	0.84 ± 0.06	0.84 ± 0.05	0.84 ± 0.08	0.911
TBW (kg)	35.16 ± 7.88	30.76 ± 3.88	42.77 ± 7.22	<0.001 *
BFM (kg)	16.22 ± 9.18	17.40 ± 7.75	14.17 ± 10.97	0.004 *
FFM (kg)	48.00 ± 10.77	42.03 ± 5.34	58.35 ± 9.95	<0.001 *
SMM (kg)	26.54 ± 6.56	22.87 ± 3.19	32.89 ± 6.02	<0.001 *
PBF (%)	24.51 ± 9.50	28.24 ± 7.17	18.05 ± 9.59	<0.001 *

X—mean; SD—standard deviation; BMI—body mass index; WHR—waist-to-hip ratio; TBW—total body water; BFM—body fat mass; FFM—fat-free mass; SMM—skeletal muscle mass; PBF—percentage body fat. * = *p* < 0.05—statistically significant differences between sexes (independent samples *t*-test or Mann–Whitney U test, as appropriate).

**Figure 1:**
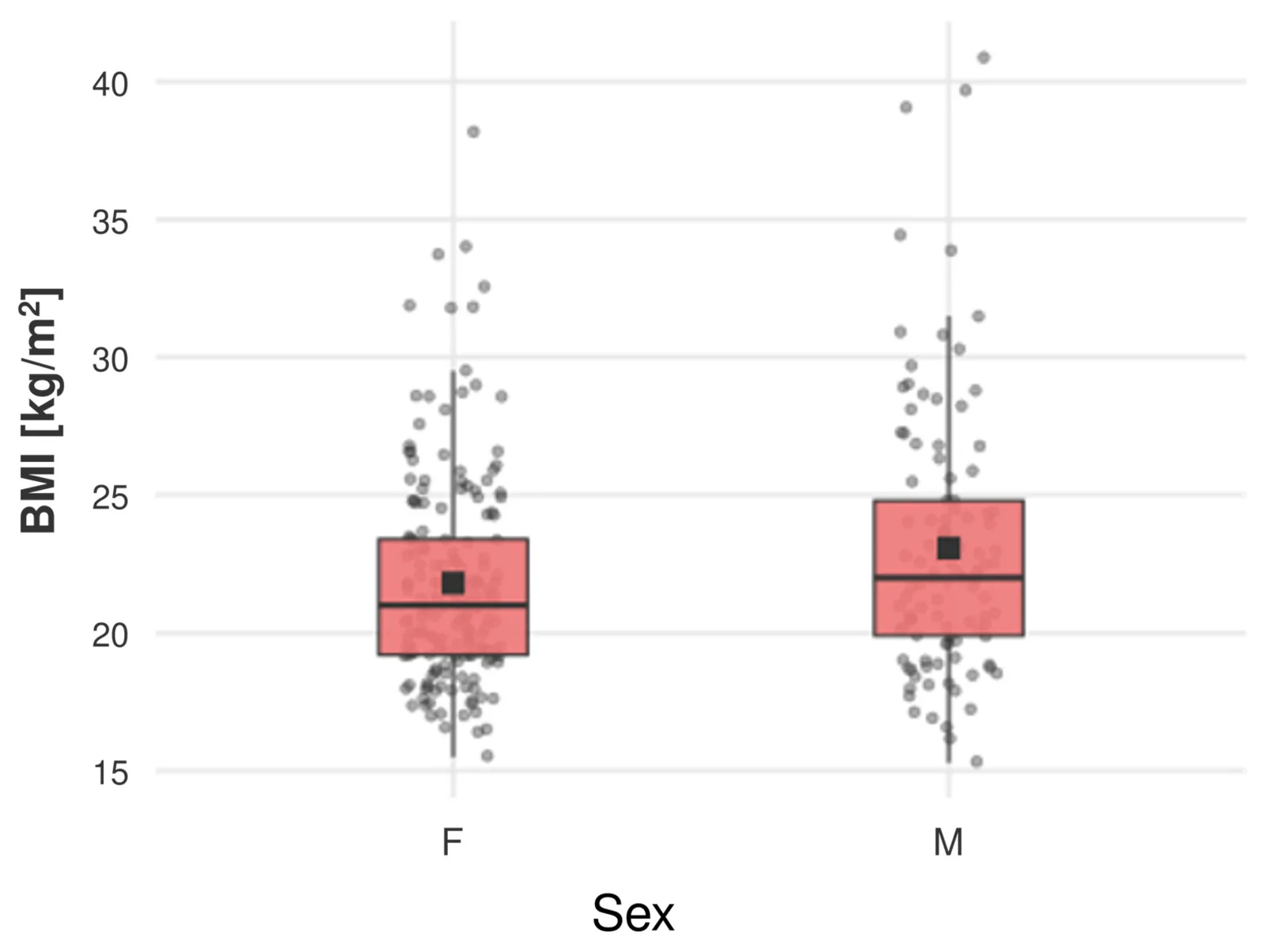
Distribution of body mass index (BMI) by sex.

The sex-specific distribution of BMI is presented in Figure 1. The descriptive distribution was shifted upward among males, consistent with the between-sex comparison in Table 1.

Sex-specific differences in DOS and SATAQ-3 scores are presented in Table 2. Females had higher mean DOS and pressure/internalization scores than males, whereas athletic internalization, information seeking, and Information did not differ significantly. Mean DOS scores in both groups were below the proposed screening threshold of 25. Because SATAQ-3 measurement invariance across sexes was not tested, these differences should not be interpreted as evidence that females are intrinsically more susceptible to sociocultural influence.

SATAQ-3 subscale scores were treated as continuous indicators of appearance-related sociocultural pressure, internalization, and information use rather than categorical measures.

**Table 2:** Orthorexia symptoms (DOS) and sociocultural appearance internalization (SATAQ-3) by sex.

Variable	Females (*n* = 182)	Males (*n* = 105)	*p*-Value
DOS score	20.81 ± 6.66	19.15 ± 5.72	0.033 *
SATAQ pressure/internalization	31.30 ± 13.29	24.73 ± 11.76	<0.001 *
SATAQ athletic internalization	10.67 ± 4.60	10.03 ± 5.05	0.273
SATAQ information seeking	20.43 ± 5.67	19.87 ± 5.69	0.416
SATAQ information	14.04 ± 5.80	12.94 ± 5.90	0.125

X—mean; SD—standard deviation; * = *p* < 0.05.

Differences in SATAQ-3 scores across DOS screening categories are shown in Table 3. The category labels describe score ranges only and do not indicate clinically diagnosed orthorexia.

Of the 287 participants, 226 (78.7%) scored below 25, 36 (12.5%) scored 25–29, and 25 (8.7%) scored 30 or higher. Thus, 21.3% scored at or above the proposed risk threshold; this proportion should not be interpreted as disorder prevalence because the cutoffs have not been validated specifically in Polish adolescents.

Participants in both higher-score categories had higher pressure/internalization and athletic internalization scores than those scoring below 25 (all Holm-adjusted *p* < 0.01), while the two higher-score categories did not differ significantly on these subscales.

For the Information subscale, all three screening categories differed: scores were lowest below 25, higher at 25–29, and highest at 30 or above (all Holm-adjusted *p* < 0.05).

Information-seeking scores did not differ significantly across screening categories (Kruskal-Wallis *p* = 0.538).

**Table 3:** SATAQ-3 subscales across DOS screening categories.

SATAQ-3 Subscale	DOS < 25	DOS 25–29	DOS ≥ 30	*p* Value
	(*n* = 226)	(*n* = 36)	(*n* = 25)	
Pressure/internalization	26.52 ± 11.79 ^a^	35.56 ± 13.99 ^b^	40.76 ± 13.93 ^b^	<0.001
Athletic internalization	9.73 ± 4.63 ^a^	13.33 ± 4.41 ^b^	12.68 ± 4.30 ^b^	<0.001
Information seeking	20.14 ± 5.82	20.86 ± 5.35	20.08 ± 4.89	0.538
Information	12.66 ± 5.27 ^a^	15.89 ± 6.36 ^b^	19.28 ± 6.23 ^c^	<0.001

X = mean; SD = standard deviation. Values with different superscript letters differ significantly between groups (Dunn’s post-hoc test with Holm adjustment, *p* < 0.05). DOS categories are screening-based and not diagnostic.

### 3.2. Associations and Predictors of Orthorexia Symptoms

In the full sample (*N* = 287), DOS scores correlated with pressure/internalization (Pearson r = 0.407, *p* < 0.001; Spearman rho = 0.368, *p* < 0.001), athletic internalization (r = 0.330, *p* < 0.001; rho = 0.331, *p* < 0.001), and Information (r = 0.385, *p* < 0.001; rho = 0.345, *p* < 0.001). Information seeking was not associated with DOS scores (r = 0.103, *p* = 0.081; rho = 0.094, *p* = 0.112). DOS scores were not significantly correlated with TBW, BFM, FFM, SMM, BMI, or PBF (all *p* >= 0.171). Applying the Bonferroni sensitivity threshold of *p* < 0.005 retained the three DOS-SATAQ associations and did not change the non-significant DOS associations with information seeking or body-composition measures. Other coefficients in Table 4 are exploratory.

**Table 4:** Pearson correlation matrix for psychological and body-composition variables (*N* = 287).

Variable	1	2	3	4	5	6	7	8	9	10	11
1. DOS	-										
2. Press./int.	0.407	-									
	(<0.001)										
3. Athl. int.	0.33	0.658	-								
	(<0.001)	(<0.001)									
4. Info seek.	0.103	0.151	−0.073	-							
	−0.081	−0.011	−0.216								
5. Information	0.385	0.764	0.585	0.108	-						
	(<0.001)	(<0.001)	(<0.001)	−0.068							
6. TBW	−0.080	−0.193	−0.013	−0.029	−0.129	-					
	−0.174	−0.001	−0.822	−0.629	−0.029						
7. BFM	−0.025	0.057	−0.060	0.077	−0.052	0.136	-				
	−0.677	−0.335	−0.311	−0.195	−0.377	−0.021					
8. FFM	−0.081	−0.192	−0.013	−0.028	−0.129	1	0.144	-			
	−0.171	−0.001	−0.826	−0.632	−0.028	(<0.001)	−0.015				
9. SMM	−0.080	−0.194	−0.011	−0.032	−0.131	1	0.134	1	-		
	−0.175	(<0.001)	−0.851	−0.588	−0.027	(<0.001)	−0.023	(<0.001)			
10. BMI	−0.030	−0.035	−0.029	0.043	−0.116	0.487	0.89	0.492	0.488	-	
	−0.612	−0.555	−0.626	−0.468	−0.049	(<0.001)	(<0.001)	(<0.001)	(<0.001)		
11. PBF	0.019	0.146	−0.035	0.102	0.003	−0.293	0.878	−0.285	−0.295	0.634	-
	−0.749	−0.013	−0.558	−0.085	−0.955	(<0.001)	(<0.001)	(<0.001)	(<0.001)	(<0.001)	

Cells show Pearson r with the two-sided *p* value in parentheses. DOS = Düsseldorf Orthorexia Scale; Press./int. = pressure/internalization; Athl. int. = athletic internalization; Info seek. = information seeking; TBW = total body water; BFM = body fat mass; FFM = fat-free mass; SMM = skeletal muscle mass; BMI = body mass index; PBF = percentage body fat. The matrix is exploratory; *p* < 0.001 is reported as <0.001.

In the initially planned eight-predictor regression, VIFs were 10.70 for BMI, 9.96 for PBF, and 6.94 for SMM, indicating that coefficients for these simultaneous body-composition predictors would be unstable. This model was therefore not used to interpret independent predictor effects.

The primary model included the four SATAQ−3 subscales, PBF, sex, and age and was estimated in all 287 participants. It explained 19.2% of DOS-score variance (R-squared = 0.192; adjusted R-squared = 0.172; F(7.279) = 9.49, *p* < 0.001). Pressure/internalization (B = 0.092, standardized beta = 0.190, *p* = 0.053) and Information (B = 0.176, standardized beta = 0.162, *p* = 0.060) were close to, but did not reach, the conventional *p* < 0.05 threshold. Athletic internalization, information seeking, PBF, sex, and age were not significant. Final-model VIFs ranged from 1.02 to 3.29, tolerances from 0.304 to 0.976, and the maximum condition index was 3.47, providing no indication of problematic collinearity in this specification (Table 5).

**Table 5:** Multiple linear regression predicting continuous DOS score (*N* = 287).

Predictor	B	SE	β	95% CI for B	*p*	VIF	Tolerance
Pressure/internalization	0.092	0.047	0.190	−0.001 to 0.186	0.053	3.29	0.304
Athletic internalization	0.146	0.101	0.109	−0.053 to 0.344	0.150	1.97	0.508
Information seeking	0.076	0.063	0.068	−0.048 to 0.200	0.230	1.09	0.914
Information	0.176	0.093	0.162	−0.008 to 0.360	0.060	2.54	0.394
PBF (%)	−0.038	0.043	−0.056	−0.122 to 0.047	0.380	1.41	0.710
Female sex	1.083	0.854	0.082	−0.599 to 2.764	0.206	1.45	0.692
Age (years)	0.080	0.270	0.016	−0.451 to 0.611	0.766	1.02	0.976

B = unstandardized coefficient; SE = standard error; β = standardized coefficient; CI = confidence interval; VIF = variance inflation factor. Female sex was coded 1 and male sex 0. Model R-squared = 0.192; adjusted R-squared = 0.172; F(7.279) = 9.49; *p* < 0.001; maximum condition index = 3.47.

## 4. Discussion

This study yielded three principal findings. First, continuous DOS scores showed moderate positive bivariate associations with SATAQ-3 pressure/internalization, athletic internalization, and Information, and the pattern was consistent in Spearman sensitivity analyses. Second, DOS scores were not significantly correlated with the measured body-composition indicators. Third, in the adjusted model, pressure/internalization and Information were close to but did not reach conventional statistical significance, while athletic internalization, information seeking, PBF, sex, and age were not independently associated with DOS scores. The final model explained 19.2% of DOS-score variance.

The distinction between bivariate and adjusted findings is important. Correlations indicate that greater orthorexic symptom severity co-occurred with greater appearance-related pressure, internalization, and information use, but the regression coefficients estimate associations after accounting for overlap among the SATAQ-3 dimensions and covariates. The findings therefore do not support describing any SATAQ-3 subscale as a confirmed independent predictor. They are consistent with earlier reports linking orthorexic symptoms with sociocultural internalization, body-image concerns, perfectionism, and related psychological characteristics [28,29,30,31,32].

Orthorexic concerns may focus on healthfulness, purity, food quality, and dietary control rather than appearance alone [3,32]. These concerns should be distinguished from evidence-based dietary patterns discussed in relation to health promotion and disease prevention [33]. The present results position appearance-related sociocultural influence as one possible pathway, not as a complete explanation of orthorexic symptoms or as evidence that orthorexia is simply another form of appearance-driven eating pathology. In addition, the SATAQ-3 assesses appearance-related information and does not identify exposure to nutrition-specific or healthy-eating content. An earlier study of women following health-food accounts reported an association between greater Instagram use and orthorexic symptoms [34]. The review by Vintró-Alcaraz et al. [17] provides a broader context in which idealized bodies, fitness lifestyles, orthorexic symptoms, and muscle dysmorphia can intersect, but the mechanism and direction of any relationship cannot be inferred from the present measures.

The absence of significant DOS associations with PBF or the other body-composition measures does not demonstrate that physical characteristics have no role in orthorexic behavior. It indicates only that the measured indicators were not associated with DOS scores in this sample and that PBF did not show an independent association in the selected adjusted model. The initially planned model also showed substantial collinearity among BMI, PBF, and SMM, which precluded stable interpretation of their simultaneous coefficients. Longitudinal studies incorporating perceived body image, pubertal development, and repeated body-composition measurements are needed to clarify temporal and developmental relationships.

Females had higher mean DOS and pressure/internalization scores than males, but sex was not significant in the adjusted model. These descriptive differences require caution because SATAQ-3 measurement invariance across sexes was not tested and the instrument may represent thin-ideal pressures more fully than muscularity-oriented pressures. The results therefore cannot establish that females are more susceptible to sociocultural influence. Pubertal stage was not assessed and may confound associations involving chronological age, sex, and body composition; specific hormonal mechanisms were not examined.

Digital-context concepts may help frame future research, but they were not tested here. Appearance-related social media consciousness concerns ongoing awareness of evaluation by an online audience and has been associated with body surveillance, shame, self-objectification, situational self-awareness, and appearance comparison [22]. The identity-bubble framework further emphasizes social identification, homophily, and information bias in early adolescents’ online networks [23]. Future studies could measure these constructs alongside nutrition-specific content exposure to distinguish active social and identity processes from the general appearance-related information captured by SATAQ-3.

The cross-sectional design also permits reverse or bidirectional explanations: adolescents with more severe orthorexic symptoms may attend more closely to appearance-related messages, and sociocultural pressure may contribute to symptom development. The observed associations cannot identify the direction of influence. Strategies to mitigate social-media-related harms to adolescent body image and disordered eating have been discussed in the literature [35]. Accordingly, media-literacy, body-image, school, or family interventions should be considered hypotheses for future prevention research rather than interventions shown by the present study to reduce orthorexic symptoms.

### Strengths and Limitations

The study combined validated symptom and sociocultural questionnaires with standardized BIA measurements in a sample of 287 adolescents. Analyses used the continuous DOS score for association testing, checked Pearson findings with Spearman correlations, corrected post-hoc group comparisons using Holm’s method, evaluated multiplicity with a Bonferroni sensitivity threshold, and formally assessed regression collinearity.

Several limitations should be considered. The cross-sectional design precludes causal inference. Convenience sampling from selected schools in one region of Poland and a 66.1% final analytic proportion limit generalizability and may introduce selection bias. Self-reported sociocultural attitudes and eating-related cognitions may be affected by response bias. No prospective a priori power analysis was conducted; the later G*Power calculation only shows that the achieved sample exceeded a conventional planning threshold.

The proposed DOS cutoffs were developed in adults and have not been validated specifically in Polish adolescents; category-based results are therefore screening descriptions rather than estimates of diagnosed disorder prevalence. The SATAQ-3 may not capture male muscularity-oriented pressures equivalently, and measurement invariance across sexes was not tested. These measurement limitations restrict interpretation of the category and sex comparisons.

Pubertal maturation was not assessed. Variation in biological development may influence body-fat distribution, skeletal muscle mass, body image, and eating behavior independently of chronological age. The study cannot evaluate specific hormonal pathways because hormone levels were not measured.

The number of comparisons increases the risk of false-positive findings. Pairwise category comparisons were Holm-adjusted, while the correlation matrix remained exploratory. A Bonferroni sensitivity threshold of *p* < 0.005 retained the DOS associations with pressure/internalization, athletic internalization, and Information and left information seeking and all measured body-composition correlations with DOS non-significant; nevertheless, replication is required.

The initially planned simultaneous inclusion of BMI, PBF, and SMM produced substantial collinearity, so PBF was retained as a single adiposity indicator in the primary model. Although final-model diagnostics were acceptable, selecting one body-composition measure may not capture other physical dimensions. Correlations among SATAQ-3 subscales also require individual coefficients to be interpreted cautiously.

Finally, the study did not measure perceived body image, perfectionism, anxiety, personality, family eating patterns, nutrition-specific social-media exposure, appearance-related social media consciousness, or identity-bubble processes. These factors may confound or explain additional variance and should be included in longitudinal, developmentally sensitive models.

These limitations constrain the interpretation of both the statistical associations and their possible practical relevance.

## 5. Conclusions

In this sample of Polish adolescents, DOS scores were positively correlated with appearance-related pressure/internalization, athletic internalization, and Information, and these bivariate associations were robust in rank-correlation sensitivity analyses. In the adjusted model, however, pressure/internalization and Information did not reach the conventional significance threshold, and PBF, sex, age, athletic internalization, and information seeking were not independently associated with DOS scores.

The findings identify appearance-related sociocultural variables as relevant correlates of orthorexic symptom severity without establishing causal mechanisms or showing that body composition has no role. DOS category results remain screening-based, and sex comparisons are limited by the absence of SATAQ-3 measurement-invariance testing. Longitudinal research using adolescent-validated orthorexia measures, developmentally sensitive assessments, and direct measures of nutrition-specific digital exposure is needed before preventive recommendations can be evaluated.

## Data Availability

The original contributions presented in this study are included in the article. Further inquiries can be directed to the corresponding author.
